# Supplementary material for: Resolving the full spectrum of human genome variation using Linked-Reads
Source: Genome Res. 2019 Apr;29(4):635–45. doi: 10.1101/gr.234443.118 (PMC6442396; doi:10.1101/gr.234443.118)
Supplement: Supplemental Material [file supp_gr.234443.118_Supplemental_longranger-2.2.zip › longranger-2.2.tar.bz2!/lr2.2-public/lib/stan/cmdstan/stan/doc/api/html/program__grammar__def_8hpp_source.html]

Stan: src/stan/gm/grammars/program\_grammar\_def.hpp Source File


|  |  |
| --- | --- |
|  | Stan  2.5.0  probability, sampling & optimization |


- Main Page
- Related Pages
- Namespaces
- Classes
- Files

- File List
- File Members

All Classes Namespaces Files Functions Variables Typedefs Enumerations Enumerator Friends Macros Pages

- src
- stan
- gm
- grammars

program\_grammar\_def.hpp

Go to the documentation of this file.

1 #ifndef STAN\_\_GM\_\_PARSER\_\_PROGRAM\_GRAMMAR\_DEF\_\_HPP

2 #define STAN\_\_GM\_\_PARSER\_\_PROGRAM\_GRAMMAR\_DEF\_\_HPP

3

4 #include <cstddef>

5 #include <iomanip>

6 #include <iostream>

7 #include <istream>

8 #include <map>

9 #include <set>

10 #include <sstream>

11 #include <string>

12 #include <utility>

13 #include <vector>

14 #include <stdexcept>

15

16 #include <boost/format.hpp>

17 #include <boost/spirit/include/qi.hpp>

18 #include <boost/lexical\_cast.hpp>

19 #include <boost/fusion/include/adapt\_struct.hpp>

20 #include <boost/fusion/include/std\_pair.hpp>

21 #include <boost/config/warning\_disable.hpp>

22 #include <boost/spirit/include/qi\_numeric.hpp>

23 #include <boost/spirit/include/phoenix\_core.hpp>

24 #include <boost/spirit/include/phoenix\_function.hpp>

25 #include <boost/spirit/include/phoenix\_fusion.hpp>

26 #include <boost/spirit/include/phoenix\_object.hpp>

27 #include <boost/spirit/include/phoenix\_operator.hpp>

28 #include <boost/spirit/include/phoenix\_stl.hpp>

29 #include <boost/spirit/include/support\_multi\_pass.hpp>

30 #include <boost/tuple/tuple.hpp>

31 #include <boost/variant/apply\_visitor.hpp>

32 #include <boost/variant/recursive\_variant.hpp>

33

34 #include <boost/spirit/include/version.hpp>

35 #include <boost/spirit/home/support/iterators/line\_pos\_iterator.hpp>

36

37 #include <stan/gm/ast.hpp>

38 #include <stan/gm/grammars/whitespace\_grammar.hpp>

39 #include <stan/gm/grammars/expression\_grammar.hpp>

40 #include <stan/gm/grammars/var\_decls\_grammar.hpp>

41 #include <stan/gm/grammars/statement\_grammar.hpp>

42 #include <stan/gm/grammars/program\_grammar.hpp>

43 #include <stan/gm/grammars/functions\_grammar.hpp>

44

45 namespace {

46  // hack to pass pair into macro below to adapt; in namespace to hide

47  struct DUMMY\_STRUCT {

48  typedef std::pair<std::vector<stan::gm::var\_decl>,

49  std::vector<stan::gm::statement> > type;

50  };

51 }

52

53

54 BOOST\_FUSION\_ADAPT\_STRUCT(stan::gm::program,

55  (std::vector<stan::gm::function\_decl\_def>, function\_decl\_defs\_)

56  (std::vector<stan::gm::var\_decl>, data\_decl\_)

57  (DUMMY\_STRUCT::type, derived\_data\_decl\_)

58  (std::vector<stan::gm::var\_decl>, parameter\_decl\_)

59  (DUMMY\_STRUCT::type, derived\_decl\_)

60  (stan::gm::statement, statement\_)

61  (DUMMY\_STRUCT::type, generated\_decl\_) )

62

63

64 namespace stan {

65

66  namespace gm {

67

68  struct add\_lp\_var {

69  template <typename T>

70  struct result { typedef void type; };

71  void operator()(variable\_map& vm) const {

72  vm.add("lp\_\_",

73  base\_var\_decl("lp\_\_",std::vector<expression>(),DOUBLE\_T),

74  local\_origin); // lp acts as a local where defined

75  }

76  };

77  boost::phoenix::function<add\_lp\_var> add\_lp\_var\_f;

78

79  struct remove\_lp\_var {

80  template <typename T>

81  struct result { typedef void type; };

82  void operator()(variable\_map& vm) const {

83  vm.remove("lp\_\_");

84  }

85  };

86  boost::phoenix::function<remove\_lp\_var> remove\_lp\_var\_f;

87

88  struct program\_error {

89  template <typename T1, typename T2, typename ,

90  typename T4, typename T5, typename T6, typename T7>

91  struct result { typedef void type; };

92

93  template <class Iterator, class I>

94  void operator()(

95  Iterator \_begin,

96  Iterator \_end,

97  Iterator \_where,

98  I const& \_info,

99  std::string msg,

100  variable\_map& vm,

101  std::stringstream& error\_msgs) const {

102

103  using boost::phoenix::construct;

104  using boost::phoenix::val;

105  using boost::spirit::get\_line;

106  using boost::format;

107  using std::setw;

108

109  error\_msgs << msg << std::endl;

110

111  size\_t idx\_errline = 0;

112  idx\_errline = get\_line(\_where);

113

114  if (idx\_errline > 0) {

115  error\_msgs << "ERROR at line " << idx\_errline << std::endl;

116

117  std::basic\_stringstream<char> sprogram;

118  sprogram << boost::make\_iterator\_range (\_begin, \_end);

119

120  // show error in context 2 lines before, 1 lines after

121  size\_t idx\_errcol = 0;

122  idx\_errcol = get\_column(\_begin,\_where) - 1;

123

124  std::string lineno = "";

125  format fmt\_lineno("% 3d: ");

126

127  std::string line\_2before = "";

128  std::string line\_before = "";

129  std::string line\_err = "";

130  std::string line\_after = "";

131

132  size\_t idx\_line = 0;

133  size\_t idx\_before = idx\_errline - 1;

134  if (idx\_before > 0) {

135  // read lines up to error line, save 2 most recently read

136  while (idx\_before > idx\_line) {

137  line\_2before = line\_before;

138  std::getline(sprogram,line\_before);

139  idx\_line++;

140  }

141  if (line\_2before.length() > 0) {

142  lineno = str(fmt\_lineno % (idx\_before - 1) );

143  error\_msgs << lineno << line\_2before << std::endl;

144  }

145  lineno = str(fmt\_lineno % idx\_before);

146  error\_msgs << lineno << line\_before << std::endl;

147  }

148

149  std::getline(sprogram,line\_err);

150  lineno = str(fmt\_lineno % idx\_errline);

151  error\_msgs << lineno << line\_err << std::endl

152  << setw(idx\_errcol + lineno.length()) << "^" << std::endl;

153

154  if (!sprogram.eof()) {

155  std::getline(sprogram,line\_after);

156  lineno = str(fmt\_lineno % (idx\_errline+1));

157  error\_msgs << lineno << line\_after << std::endl;

158  }

159  }

160  error\_msgs << std::endl;

161  }

162  };

163  boost::phoenix::function<program\_error> program\_error\_f;

164

165  template <typename Iterator>

166  program\_grammar<Iterator>::program\_grammar(const std::string& model\_name)

167  : program\_grammar::base\_type(program\_r),

168  model\_name\_(model\_name),

169  var\_map\_(),

170  error\_msgs\_(),

171  expression\_g(var\_map\_,error\_msgs\_),

172  var\_decls\_g(var\_map\_,error\_msgs\_),

173  statement\_g(var\_map\_,error\_msgs\_),

174  functions\_g(var\_map\_,error\_msgs\_) {

175

176  using boost::spirit::qi::eps;

177  using boost::spirit::qi::lit;

178

179  // add model\_name to var\_map with special origin and no

180  var\_map\_.add(model\_name,

181  base\_var\_decl(),

182  model\_name\_origin);

183

184  program\_r.name("program");

185  program\_r

186  %= -functions\_g

187  > -data\_var\_decls\_r

188  > -derived\_data\_var\_decls\_r

189  > -param\_var\_decls\_r

190  // scope lp\_\_ to "transformed params" and "model" only

191  > eps[add\_lp\_var\_f(boost::phoenix::ref(var\_map\_))]

192  > -derived\_var\_decls\_r

193  > model\_r

194  > eps[remove\_lp\_var\_f(boost::phoenix::ref(var\_map\_))]

195  > -generated\_var\_decls\_r

196  ;

197

198  model\_r.name("model declaration");

199  model\_r

200  %= lit("model")

201  > statement\_g(true,local\_origin,false) // assign only to locals

202  ;

203

204  data\_var\_decls\_r.name("data variable declarations");

205  data\_var\_decls\_r

206  %= lit("data")

207  > lit('{')

208  > var\_decls\_g(true,data\_origin) // +constraints

209  > lit('}');

210

211  derived\_data\_var\_decls\_r.name("transformed data block");

212  derived\_data\_var\_decls\_r

213  %= ( lit("transformed")

214  >> lit("data") )

215  > lit('{')

216  > var\_decls\_g(true,transformed\_data\_origin) // -constraints

217  > \*statement\_g(false,transformed\_data\_origin,false) // -sampling

218  > lit('}');

219

220  param\_var\_decls\_r.name("parameter variable declarations");

221  param\_var\_decls\_r

222  %= lit("parameters")

223  > lit('{')

224  > var\_decls\_g(true,parameter\_origin) // +constraints

225  > lit('}');

226

227  derived\_var\_decls\_r.name("derived variable declarations");

228  derived\_var\_decls\_r

229  %= ( lit("transformed")

230  >> lit("parameters") )

231  > lit('{')

232  > var\_decls\_g(true,transformed\_parameter\_origin) // -constraints

233  > \*statement\_g(false,transformed\_parameter\_origin,false) // -sampling

234  > lit('}');

235

236  generated\_var\_decls\_r.name("generated variable declarations");

237  generated\_var\_decls\_r

238  %= lit("generated")

239  > lit("quantities")

240  > lit('{')

241  > var\_decls\_g(true,derived\_origin) // -constraints

242  > \*statement\_g(false,derived\_origin,false) // -sampling

243  > lit('}');

244

245  using boost::spirit::qi::on\_error;

246  using boost::spirit::qi::rethrow;

247  using namespace boost::spirit::qi::labels;

248

249  on\_error<rethrow>(

250  program\_r,

251  program\_error\_f(

252  \_1, \_2, \_3, \_4 ,

253  "",

254  boost::phoenix::ref(var\_map\_),

255  boost::phoenix::ref(error\_msgs\_)

256  )

257  );

258  }

259

260  }

261 }

262

263 #endif

program\_grammar.hpp

stan::gm::statement

**Definition:** ast.hpp:634

stan::gm::data\_origin

const int data\_origin

**Definition:** ast.hpp:413

stan::gm::parameter\_origin

const int parameter\_origin

**Definition:** ast.hpp:415

stan::gm::DOUBLE\_T

const int DOUBLE\_T

**Definition:** ast.hpp:66

stan::gm::local\_origin

const int local\_origin

**Definition:** ast.hpp:418

BOOST\_FUSION\_ADAPT\_STRUCT

BOOST\_FUSION\_ADAPT\_STRUCT(stan::gm::program,(std::vector< stan::gm::function\_decl\_def >, function\_decl\_defs\_)(std::vector< stan::gm::var\_decl >, data\_decl\_)(DUMMY\_STRUCT::type, derived\_data\_decl\_)(std::vector< stan::gm::var\_decl >, parameter\_decl\_)(DUMMY\_STRUCT::type, derived\_decl\_)(stan::gm::statement, statement\_)(DUMMY\_STRUCT::type, generated\_decl\_)) namespace stan

**Definition:** program\_grammar\_def.hpp:54

functions\_grammar.hpp

expression\_grammar.hpp

statement\_grammar.hpp

stan::gm::program

**Definition:** ast.hpp:795

stan::gm::derived\_origin

const int derived\_origin

**Definition:** ast.hpp:417

var\_decls\_grammar.hpp

stan::gm::model\_name\_origin

const int model\_name\_origin

**Definition:** ast.hpp:412

ast.hpp

stan::gm::transformed\_data\_origin

const int transformed\_data\_origin

**Definition:** ast.hpp:414

whitespace\_grammar.hpp

stan::gm::transformed\_parameter\_origin

const int transformed\_parameter\_origin

**Definition:** ast.hpp:416


---

[ Stan Home Page ]

*© 2011–2014,
Stan Development Team.*
